# *ALK*阳性非小细胞肺癌靶向治疗耐药机制及预后标志物的研究进展

**DOI:** 10.3779/j.issn.1009-3419.2020.101.44

**Published:** 2020-11-20

**Authors:** 莎莎 王, 远凯 石, 晓红 韩

**Affiliations:** 1 100021 北京，国家癌症中心，国家肿瘤临床医学研究中心，中国医学科学院北京协和医学院肿瘤医院检验科 Department of Clinical Laboratory, National Cancer Center, National Clinical Research Center for Cancer, Cancer Hospital, Chinese Academy of Medical Sciences & Peking Union Medical College, Beijing Key Laboratory of Clinical Study on Anticancer Molecular Targeted Drugs, Beijing 100021, China; 2 100021 北京，国家癌症中心，国家肿瘤临床医学研究中心，中国医学科学院北京协和医学院肿瘤医院内科，抗肿瘤分子靶向药物临床研究北京市重点实验室 Department of Medical Oncology, National Cancer Center, National Clinical Research Center for Cancer, Cancer Hospital, Chinese Academy of Medical Sciences & Peking Union Medical College, Beijing Key Laboratory of Clinical Study on Anticancer Molecular Targeted Drugs, Beijing 100021, China; 3 100032 北京，中国医学科学院，北京协和医学院，北京协和医院临床药理研究中心 Clinical Pharmacology Research Center, Peking Union Medical College Hospital, Chinese Academy of Medical Sciences & Peking Union Medical College, Beijing 100032, China

**Keywords:** 间变性淋巴瘤激酶, 肺肿瘤, ALK抑制剂, 耐药, 预后标志物, Anaplastic lymphoma kinase, Lung neoplasms, ALK inhibitors, Drug resistance, Prognostic markers

## Abstract

棘皮动物微管相关类蛋白4-间变性淋巴瘤激酶(echinoderm microtubule-associated protein like 4-anaplastic lymphoma kinase, *EML4-ALK*)融合占非小细胞肺癌(non-small cell lung cancer, NSCLC)患者的3%-5%。随着对该驱动基因的深入研究，以Crizotinib为代表的ALK抑制剂逐渐被开发并应用于临床。然而，不同患者对ALK靶向治疗的反应存在差异，且多数ALK靶向治疗患者最终会不可避免地出现耐药，导致肿瘤进展。利用预后标志物监测患者疗效及时改变治疗方案，以及根据耐药机制选择个体化的后续治疗，可以有效地改善患者的预后。本文将对ALK抑制剂的耐药机制以及相关的预后标志物展开综述，探讨ALK靶向治疗疗效预测以及耐药患者后续治疗方案的选择。

## Abstract (en)

Echinoderm microtubule-associated protein like 4-anaplastic lymphoma kinase (*EML4-ALK*) fusion accounts for 3%-5% of non-small cell lung cancer (NSCLC) patients. With the in-depth study of the *EML4-ALK* driver gene, ALK inhibitors represented by crizotinib have been gradually developed and applied in the clinic. However, the response to ALK-targeted therapy is heterogeneous among different patients. Most patients with ALK-targeted therapy will inevitably develop drug resistance, leading to tumor progression. Monitoring the efficacy of patients with prognostic markers to change the treatment in time, and selecting individualized follow-up treatment according to the mechanism of drug resistance, can effectively improve the prognosis of patients. This article will review the mechanism of ALK tyrosine kinase inhibitor (ALK-TKI) resistance and related prognostic markers to discuss the prediction for ALK-targeted therapy and the choice of subsequent treatment for drug-resistant patients.

肺癌是我国发病率和死亡率最高的恶性肿瘤^[1]^，其中以非小细胞肺癌(non-small cell lung cancer, NSCLC)最为常见，约占85%。肺癌早期发病隐匿，多数NSCLC患者诊断时已是晚期，预后不佳。间变性淋巴瘤激酶(anaplastic lymphoma kinase, *ALK*)基因与棘皮动物微管相关类蛋白4(echinoderm microtubule-associated protein like 4, *EML4*)基因由于染色体倒位而发生融合，形成具有强大致癌作用的融合基因*EML4-ALK*，促使细胞发生癌变，约占NSCLC患者的3%-5%^[2]^。随着肿瘤分子靶向治疗的进展，已有多种ALK酪氨酸激酶抑制剂(ALK tyrosine kinase inhibitors, ALK-TKI)逐渐应用于*EML4-ALK*阳性的NSCLC患者，较传统化疗显示出更好的疗效。Crizotinib(克唑替尼)是首个被美国食品药品监督管理局(Food and Drug Administration, FDA)批准用于*ALK*阳性NSCLC患者治疗的ALK-TKI^[3]^。随后，抑制能力更强、血脑屏障通透性更高的二代ALK抑制剂Ceritinib(色瑞替尼)、Alectinib(艾乐替尼)和Brigatinib(布加替尼)以及三代ALK抑制剂Lorlatinib(劳拉替尼)等应运而生。然而，由于遗传背景和机体状况不同，不同患者对ALK靶向治疗的反应存在差异。虽然目前已发现较多NSCLC化疗和免疫治疗相关的预后标志物，ALK靶向治疗预后标志物却少有报道。此外，大多数患者用药1年后会不可避免地出现耐药，导致疾病进展。明确患者的耐药机制并据此选择针对性的药物进行后续治疗十分重要。本文将对ALK抑制剂的耐药机制以及相关的预后标志物展开综述，为*ALK*阳性NSCLC患者靶向治疗疗效预测和耐药后治疗方案选择提供方向。

## 1 ALK抑制剂的耐药机制

虽然ALK-TKI在*ALK*阳性NSCLC患者中取得了较好的疗效，但大部分患者最终都不可避免地会产生耐药，出现脑或肝等其他部位的转移。ALK-TKI靶向治疗耐药分为原发性耐药和获得性耐药。原发性耐药患者较少，潜在的耐药机制暂不明确。多数患者属于获得性耐药，获得性耐药机制主要分为药物靶点变异(包括*ALK*拷贝数扩增或激酶区突变)、旁路激活及其他耐药机制。目前已报道的耐药机制汇总见表 1，部分患者多种耐药机制共存。

**表 1:** *ALK*阳性非小细胞肺癌靶向治疗的耐药机制 The drug resistance mechanisms of targeted therapy in *ALK* -positive non-small cell lung cancer

Drug	Drug resistance mechanisms			
	*ALK* mutation	*ALK* amplification	Bypass track activation	Other mechanisms
Crizotinib	L1196M, C1156Y/S, G1269A, S1206Y/F, L1152R, F1174C/I/V/L, G1128A, 1151Tins, E1210K, G1202R, I1171T, F1245V, I1268L, G1202R+G1269A, I1171T+I1268V+E1210K	CNG	EGFR activation; HER2/3 activation; IGF-1R activation; Src activation; *KRAS* G12C, G12V, Q22K mutation; Autophagy activation; Raf-1, P2Y activation	EMT; Histologic transformation; Hypoxia
	L1152R+EGFR activation; 1151Tins+EGFR activation; G1202R+KIT amplification; G1202R+IGF-1R activation; L1196M+EMT	CNG+L1196M; CNG +G1269A; CNG+1151Tins; CNG+EGFR L858R	EGFR activation+KIT amplification; EGFR L858R+Loss of *ALK* gene	Loss of *ALK* gene; P-gp overexpression; *POLE* mutation; Histone acetylation, miR-449 and miR-34a decrease; PD-L1 overexpression; exosome RNA
Ceritinib	C1156Y, G1202R, F1174L/C/V, V1180L, L1196M, G1202del, D1203N, I1171N, T1151M	CNG	EGFR activation; HER3 activation	(1) EMT; (2) P-gp overexpression; PD-L1 overexpression; Histone acetylation, miR-449 and miR-34a decrease; exosome RNA; miR-100-5p overexpression
Alectinib	V1180L, I1171N/S/T, L1196M/Q, G1202R, F1174I/V, V1185L+L1196M		EGFR activation; HER3 activation; IGF-1R activation; MET activation; Src activation	(1) EMT; (2) NMU overexpression; PD-L1 overexpression
Brigatinib	G1202R, D1203N, E1210K, S1206Y/C	-	-	-
Lorlatinib	L1198F/M, G1202R, D1203N, F1174C/L, I1171X, G1269A		EGFR activation	(1) EMT; (2) *NF2* loss-of-function mutations; miR-100-5p overexpression
	I1171S+G1269A, I1171N+L1198F/D1203N, G1202R+G1269A/ L1196M/T1151M/F1174L/C, C1156Y+L1198F, G1202R+L1204V+G1269A, E1210K+D1203N+G1269A			
ALK: anaplastic lymphoma kinase; CNG: copy number gain; EGFR: epidermal growth factor receptor; HER: human epidermal growth factor receptor; IGF-1R: insulin-like growth factor 1 receptor; Src: sarcoma gene; KRAS: kirsten rat sarcoma viral oncogene homolog; EMT: epithelial to mesenchymal transition; KIT: v-kit Hardy-Zuckerman 4 feline sarcoma viral oncogene homolog; P-gp: P-glycoprotein; PD-L1: programmed cell death ligand 1; NMU: neuromedin.				

### 1.1 原发性耐药

研究^[4]^表明Crizotinib原发耐药约占*ALK*阳性NSCLC患者的6.5%，患者在开始Crizotinib治疗后立即出现疾病进展，中位无进展生存期(progression-free survival, PFS)仅为1.2个月，但未发现其临床特征(例如腺癌组织学类型、吸烟史、年龄等)与其他患者存在显著差异。由于原发性耐药患者较为罕见，尚缺少相关耐药机制的研究。多个病例报告表明，*ALK*阳性NSCLC患者原发耐药可能与*ALK*突变^[5]^、*MYC*基因扩增^[6]^、*EGFR*共突变^[7]^、*KRAS*共突变^[8]^、*Bim*基因缺失多态性^[9]^以及*EML4-ALK*重排突变等位基因分数(mutant allele fraction, MAF)较低^[10]^等有关，后续仍需要扩大样本量对这些机制进行更深入的探究。

### 1.2 获得性耐药

#### 1.2.1 *ALK*激酶区突变

*ALK*激酶结构域突变是最常见的ALK-TKI耐药机制之一，约占Crizotinib耐药机制的30%^[11]^。Choi等^[12]^首次报道在Crizotinib耐药的NSCLC患者中发现L1196M和C1156Y突变，随后1151Tins、L1152R、G1202R、S1206Y和G1269A突变相继被发现^[13]^。这些*ALK*突变散布在激酶结构域的各个区域影响其功能，包括溶剂暴露区(G1202R, S1206Y)、管家残基区(L1196M)、ATP结合区(G1269A)和αC-螺旋N末端(1151Tins, L1152R, C1156Y)^[13]^。此外，Sasaki等^[14]^首先在炎性肌纤维母细胞瘤患者中发现*ALK* F1174L突变作为潜在的Crizotinib耐药机制，随后在Crizotinib耐药的*ALK*阳性NSCLC患者中也发现该突变^[15]^。其他Crizotinib耐药相关的*ALK*突变还包括G1128A、I1171T、E1210K、C1156S和F1245V等^[16-19]^。尽管多数Crizotinib耐药患者表现为单一*ALK*突变，仍有研究发现少数耐药患者出现多重*ALK*突变(≥2)，例如G1202R+G1269A共突变^[15]^。

为了克服Crizotinib耐药，新一代的ALK-TKI逐渐被开发并应用于临床。但随着疾病的进展，多数患者会再次出现新的*ALK*突变进而耐药。与Crizotinib相比，二代ALK-TKI治疗更易出现ALK耐药性突变。其中，G1202R是接受二代ALK-TKI治疗患者最常见的耐药突变^[16]^。除此之外，Ceritinib耐药突变还包括F1174L/C/V、L1196M、G1202del、D1203N和T1151M以及多重*ALK*突变C1156Y+I1171N等^[16, 18, 20]^。Katayama等^[21]^首先在Alectinib耐药细胞系和患者组织中发现V1180L和I1171T耐药突变。随后陆续发现I1171N/S和L1196M突变也参与Alectinib耐药^[16]^。除G1202R外，Brigatinib耐药患者组织中还检测到D1203N、S1206Y/C和E1210K耐药突变^[16]^。Lorlatinib对上述耐药突变均有较好的抑制能力，常用于两种或多种ALK-TKI治疗失败患者。Shaw等^[22]^发现ALK C1156Y突变的Crizotinib耐药患者经Lorlatinib治疗后耐药，出现新的L1198F突变。与一代和二代ALK-TKI不同，多数Lorlatinib耐药患者表现为多重*ALK*突变。研究^[19, 23-26]^表明，三代ALK-TKI Lorlatinib耐药患者中多重*ALK*突变所占比例约为二代ALK-TKI耐药患者的2倍，主要包括G1269A+I1171S/C1156Y/G1202R、G1202R+L1196M/F1174L、L1196M+D1203N等类型。

#### 1.2.2 *ALK*融合基因拷贝数增加

*ALK*融合基因扩增会导致Crizotinib无法完全抑制下游信号，是肿瘤进展的另一重要原因，约占Crizotinib耐药患者的15%^[27]^。Katayama等^[28]^首次在Crizotinib诱导耐药的H3122细胞系(H3122CR)中发现野生型EML4-ALK拷贝数增加，同时合并有*ALK* L1196M突变。随后Doebele等^[29]^在Crizotinib耐药患者中发现2例*ALK*基因拷贝数增加，其中1例合并有*ALK* G1269A突变，进一步表明*ALK*融合基因拷贝数增加与Crizotinib耐药有关。

#### 1.2.3 旁路信号通路的异常激活

信号传导途径旁路激活也是ALK-TKI耐药的机制之一，多见于经历多种ALK-TKI治疗后的患者^[15]^。表皮生长因子受体(epidermal growth factor receptor, EGFR)异常激活是最常见的旁路激活途径，约占Crizotinib耐药患者的30%^[27]^，主要通过上调EGFR及其配体的表达实现。EGFR异常激活首次发现于耐药细胞中，与EGFR配体双调蛋白及表皮生长因子(epidermal growth factor, EGF)分泌增加有关^[30]^。除EGF外，体外实验还发现EGFR的其他配体转化生长因子α(transforming growth factor-α, TGF-α)和肝素结合性表皮生长因子(heparin-binding epidermal growth factor, HB-EGF)也参与Crizotinib耐药^[31]^。随后，Katayama等^[32]^在*ALK*阳性肺癌患者临床样本中证实Crizotinib耐药后EGFR磷酸化水平升高。此外，有研究^[29, 33]^在Crizotinib耐药患者中发现*EGFR* L858R突变，提示*EGFR*突变也可能与Crizotinib耐药有关。人表皮生长因子受体2/3(human epidermal growth factor receptor 2, HER2/3)与EGFR同属HER家族，有研究^[32]^在Crizotinib耐药细胞中发现HER3配体神经调节蛋白1(neuroregulin1, NRG1)的高表达，可促进HER2和HER3的相互作用，影响下游通路导致耐药。同样地，研究^[34-36]^表明EGFR通路的异常激活也参与二代和三代ALK-TKI的耐药。

除了EGFR通路外，其他旁路信号通路的异常改变也与ALK-TKI耐药有关。研究^[29, 37]^发现，部分*ALK*阳性患者Crizotinib治疗进展后出现KRAS G12C、G12V或Q22K突变，但该突变是否直接导致Crizotinib耐药有待研究。Katayama等^[32]^发现在配体干细胞因子(stem cell factor, SCF)存在下编码酪氨酸激酶的*KIT*基因扩增会导致H3122细胞对Crizotinib产生耐药。研究^[38]^表明，H3122细胞Crizotinib耐药还与细胞自噬通路激活有关，伴随着ALK及其磷酸化蛋白表达水平的降低。体内外实验证实，使用细胞自噬抑制剂氯喹可以逆转Crizotinib耐药。Wilson等^[39]^还发现嘌呤P2Y受体在Crizotinib耐药组织中高表达，通过激活蛋白激酶C诱导Crizotinib耐药。此外，Src通路异常激活和胰岛素样生长因子1受体(insulin-like growth factor-1 receptor, IGF-1R)及其配体IGF-1过表达也参与Crizotinib和Alectinib的耐药^[40-43]^。*MET*扩增以及配体肝细胞生长因子(hepatocyte growth factor, HGF)自分泌激活MET信号通路均可以导致Alectinib失效^[35]^。

#### 1.2.4 其他耐药机制

ALK-TKI耐药的其他机制主要包括表型改变、相关基因缺失及蛋白表达异常等。上皮-间质转化(epithelial to mesenchymal transition, EMT)是最常见的组织学变化，表现为上皮组织标志物(如E-钙黏蛋白)丢失，间质组织标志物(如波形蛋白)表达增加。Kim等^[44]^对H2228细胞系进行Crizotinib诱导耐药后，细胞形态由圆形变为纺锤形，细胞迁移和侵袭能力增强，且E-钙黏蛋白和细胞角蛋白-18表达下降，波形蛋白和AXL蛋白表达增加，提示EMT可能参与Crizotinib耐药。Wei等^[45]^对Crizotinib耐药患者治疗前后组织样本进行测序，发现耐药组织突变基因富集后显示4条与EMT相关的通路，进一步证实EMT可能参与Crizotinib的耐药。Kogita等^[46]^发现缺氧能通过诱导EMT从而使H3122细胞对Crizotinib产生耐药。同样地，研究^[25, 47]^表明EMT也介导Ceritinib、Alectinib和Lorlatinib的耐药。此外，有研究^[48]^报道NSCLC患者耐药后组织学类型转化为小细胞肺癌的病例，但其分子机制未知。

有研究^[29]^发现Crizotinib耐药患者出现*ALK*基因缺失的现象，但由于*ALK*基因检测方法不稳定，无法排除假阴性结果，暂不明确*ALK*缺失是否是Crizotinib的耐药机制。Kang等^[49]^发现耐药患者存在DNA错配修复基因*POLE*突变，致使肿瘤突变负荷(tumor mutational burden, TMB)增加，可能与Crizotinib耐药有关。Recondo等^[25]^在Lorlatinib耐药患者中发现*NF2*功能缺失突变。组蛋白乙酰化与miR-449、miR-34a等表观遗传学的改变与Crizotinib、Ceritinib耐药相关^[50]^。miR-100-5p上调可能通过抑制mTOR通路相关mRNA的表达进而导致ALK-TKI耐药^[51]^。

在蛋白表达异常中，p-糖蛋白(p-glycoprotein, p-gp)过表达会影响药物转运从而导致ALK-TKI耐药^[20]^。Kim等^[52]^发现Crizotinib、Ceritinib和Alectinib耐药细胞总程序性死亡受体配体1(programmed cell death ligand 1, PD-L1)、细胞表面PD-L1和PD-L1 mRNA水平均高于亲本细胞株，且多重耐药细胞株中表达水平更高; RNA-seq结果显示ALK-TKI耐药与多个免疫相关基因的表达有关。26例患者的组织标本免疫组化检测结果证实，PD-L1的高表达可能参与ALK-TKI的耐药。此外，研究发现，来自耐药细胞的外泌体可诱导敏感株产生耐药，增加细胞迁移能力^[18]^。

### 1.3 克服ALK-TKI耐药的策略

#### 1.3.1 序贯治疗

对于Crizotinib治疗后进展的*ALK*阳性NSCLC患者，选择合适的后续治疗方案延长患者的生存时间十分重要，常见的措施即序贯使用二代、三代ALK-TKI。与化疗相比，二代TKI治疗Crizotinib耐药患者的客观缓解率(objective response rate, ORR)更高，PFS延长。ALK G1202R是二代ALK-TKI最常见的耐药突变，使用三代抑制剂Lorlatinib可以有效地克服该耐药突变^[16]^。此外，由于不同ALK-TKI的化学结构不同，Lorlatinib耐药的*ALK*突变可能会恢复对早期ALK-TKI的敏感性。Shaw等^[22]^报道了1例*ALK* C1156Y突变的Cizotinib耐药患者经Lorlatinib治疗后耐药，出现新的L1198F突变并恢复对Crizotinib的敏感性。以上研究结果表明，根据患者的*ALK*突变谱以及各ALK-TKI的敏感靶点，选择不同ALK-TKI组合进行序贯治疗，有望进一步延长患者的生存时间。临床试验^[53]^结果表明，二代和三代ALK-TKI用于一线治疗*ALK*阳性NSCLC患者的疗效优于Crizotinib，Crizotinib作为一线治疗首选药物的地位也受到了挑战。

#### 1.3.2 靶向信号通路

抑制ALK下游信号通路可以有效克服ALK-TKI耐药。MEK是ALK和受体酪氨酸激酶(receptor tyrosine kinase, RTKs)信号通路下游的关键蛋白，联合Crizotinib和MEK抑制剂Selumetinib可通过抑制下游Ras/MAPK信号通路，逆转H3122CR耐药^[54]^。抑制热休克蛋白90(heat shock protein 90, Hsp90)可以导致EML4-ALK和多种致癌信号蛋白降解，进而杀伤癌细胞。Sang等^[55]^发现Hsp90抑制剂Ganetespib在体内外实验中表现出较好的抗肿瘤活性，且对Crizotinib耐药的*ALK*阳性NSCLC患者具有一定疗效。但由于Hsp90抑制剂副作用较大，其临床应用受限。信号传导途径旁路激活是ALK-TKI耐药的重要原因，靶向抑制EGFR、MET、KIT、Src和IGF-1R等信号通路可以有效地克服耐药。例如，联合Alectinib与EGFR TKI、MET抑制剂(Crizotinib或PHA-665752)或Src抑制剂(Saracatinib)，可成功逆转Alectinib及Lorlatinib耐药^[25, 56, 57]^。此外，mTOR抑制剂可以逆转*NF2*功能缺失突变导致的Lorlatinib耐药^[25]^。HDAC抑制剂(Quisinostat)可以通过增加miR-200c的表达抑制EMT从而克服耐药^[58]^。

#### 1.3.3 其他后续治疗措施

除了上述靶向ALK以及信号通路等方式，后续治疗还包括局部治疗、全身化疗和联合抗血管靶向治疗等多方面。若患者疾病进展后无症状、仅有脑部症状或全身孤立病灶，可考虑局限病灶根治性治疗，例如立体定向烧蚀/消融放疗技术或手术。若患者出现全身多发病灶，推荐多西他赛、培美曲塞或吉西他滨等全身化疗以及联合抗血管靶向药贝伐珠单抗等进行后续治疗^[59]^。虽然免疫治疗在驱动基因阴性的NSCLC患者中显示出较好的疗效，但其对*ALK*阳性NSCLC患者的疗效尚有待商榷。Lin等^[60]^的研究表明Crizotinib治疗后患者序贯使用免疫检查点抑制剂(immune checkpoint inhibitor, ICI)治疗会出现严重的肝毒性。研究^[61]^发现，无论PD-L1表达水平高低，ICI治疗后*ALK*阳性NSCLC患者的客观缓解率为均较低，故暂不推荐使用免疫治疗作为ALK-TKI耐药后的后续治疗。

## 2 ALK-TKI的预后标志物

原发性和获得性耐药机制中的靶点改变往往意味着ALK-TKI的疗效较差，可以作为预后预测和疗效监测的生物标志物。除此之外，患者的*EML4-ALK*融合类型、基因变异情况以及多种血清标志物等也与ALK-TKI的疗效有关，可用于预测患者的预后情况，指导靶向治疗用药。

### 2.1 *ALK*融合类型

根据参与融合的*EML4*外显子的不同，*EML4-ALK*融合可分为多种不同的类型。其中，v1型和v3型是*ALK*阳性NSCLC患者中最常见的融合类型^[62]^。由于v1型融合蛋白包含部分串联非典型β-螺旋(tandem atypical β-propeller, TAPE)结构，而v3型融合蛋白缺乏该结构，故v1型融合蛋白的稳定性不如v3型，对药物的敏感性更高^[63]^。Yoshida等^[62]^发现Crizotinib治疗后，v1型患者的PFS较其他融合类型患者长。同样地，Woo等^[63]^的研究表明，v3a/b型患者经Crizotinib治疗后疗效较其他患者差。然而，Lin等^[64]^并未发现v1与v3型患者的Crizotinib疗效存在显著差异，但该研究显示Lorlatinib治疗后v3型患者预后更佳。由于*ALK*阳性NSCLC患者数量较少，*ALK*融合分型并非常规检测，各研究的样本量有限，*EML4-ALK*融合类型是否与ALK-TKI的敏感性有关，能否影响患者的预后尚有待探究。非相互/相互(non-reciprocal/reciprocal)*ALK*重排是指3’-*ALK*与*EML4*形成融合后，5’-*ALK*又与另一伴侣结合形成5’-*ALK*融合，其结构更为复杂。研究^[65]^表明，非相互/相互*ALK*重排患者较单独的*EML4-ALK*融合患者预后更差，易发生脑转移，是患者接受Crizotinib治疗的独立疗效预测标志物。

### 2.2 *ALK*突变的数量和类型

Shaw等^[66]^发现，Crizotinib耐药患者中，*ALK*突变存在与否对Lorlatinib的治疗效果没有影响; 相反，在二代ALK-TKI治疗后耐药患者中，存在*ALK*突变的患者ORR更高，PFS更长，且单一*ALK*突变患者疗效优于多重*ALK*突变患者。在该研究中，Lorlatinib对*ALK* G1202R/del突变类型的患者疗效最佳。由于不同的*ALK*突变对不同ALK抑制剂的敏感性存在差异，根据患者的突变情况选择敏感的TKI能够使患者获得更好的疗效。

### 2.3 *TP53*共突变

*TP53*突变是*ALK*阳性NSCLC患者常见的突变之一。Yu等^[15]^在Crizotinib治疗患者中发现，存在*TP53*突变的患者PFS较*TP53*野生型患者短(8个月*vs* 13个月)，提示*TP53*基因突变可能是*ALK*阳性NSCLC患者的不良预后因子。此外，在*TP53*野生型患者中，v3型融合患者疗效较非v3型患者差，但未在*TP53*突变患者中发现该差异。最近的一项*meta*分析^[67]^也表明，*TP53*突变患者经ALK-TKI治疗后的PFS和OS均较野生型患者短。

### 2.4 循环肿瘤细胞(circulating tumor cell, CTC)

基线CTC水平与NSCLC患者接受一线标准化疗后的预后有关。为探究基线CTC水平与靶向治疗的预后是否有关联，Tong等^[68]^对43例*EGFR*或*ALK*阳性患者的基线血CTC水平进行了检测，发现CTC计数 < 8 CTCs/3.2 mL的患者中位PFS和总生存期(overall survival, OS)较CTC≥8 CTCs/3.2 mL的患者长，差异具有统计学意义。但该研究仅纳入36例*EGFR*阳性和7例*ALK*阳性患者，后续需要增加*ALK*阳性样本量验证该结论。此外，Pailler等^[69]^发现存在*ALK*扩增的CTC数量下降预示着更长的PFS(14.0个月*vs* 6.1个月)，表明CTC的动态改变与患者预后有关。

### 2.5 血清蛋白质

*ALK*阳性的间变性大细胞淋巴瘤患者血液ALK自身抗体滴度与预后负相关，由于*ALK*阳性的NSCLC患者血液中也存在不同滴度的ALK自身抗体，Awad等^[70]^根据血清ALK自身抗体水平将53例*ALK*阳性NSCLC患者分为两组，发现高滴度ALK自身抗体患者预后优于低滴度患者，但由于样本量较小且治疗方式的不统一，*P*值不显著，仍需扩大样本对该差异进行验证。除了血清自身抗体，有研究^[71]^利用质谱技术对Crizotinib治疗患者的疗前血进行了血清蛋白质的筛查，发现DPP4、KIT和LUM蛋白的表达水平与患者的PFS长短有关，有望作为ALK靶向治疗的疗效标志物。

### 2.6 其他

Kwok等^[18]^发现患者由基线、疾病稳定到疾病进展的过程中，血清外泌体RNA表达的种类和含量发生动态改变，可能与*ALK*阳性肺腺癌患者预后有关。*Bim*基因缺失多态性是Crizotinib治疗的不良预后因子^[9]^。Yun等^[72]^对*ALK*阳性患者组织中的YAP(yes associated protein)蛋白表达水平进行了检测，发现部分缓解(partial response, PR)患者的YAP表达低于疾病稳定(stable disease, SD)和进展(progressive disease, PD)的患者，表明高表达的YAP可能会影响ALK-TKI的疗效。此外，血常规指标如中性粒细胞/淋巴细胞值、中性粒细胞/(白细胞-中性粒细胞)值、血小板/淋巴细胞值、白细胞数以及疗前血乳酸脱氢酶水平等都与Crizotinib的疗效存在关联^[73, 74]^。

## 3 总结

随着测序技术的发展和靶向治疗研究的深入，全面检测患者的基因改变，从而制定个性化的靶向治疗方案逐渐成为精准治疗时代指导临床用药的一个重要思路。除了传统的免疫组化、荧光原位杂交以及二代测序技术，近年来Foundation Medicine开发的基于NGS的全面基因组测序(comprehensive genomic profiling, CGP)伴随诊断试剂盒，可以同时检测实体瘤相关的三百多个基因，并且提供TMB和微卫星不稳定性(microsatellite instability, MSI)信息，指导靶向治疗和免疫治疗用药，已被FDA批准用于泛瘤种伴随诊断。与全基因组测序相比，CGP仅覆盖具有明确临床价值的基因，降低了数据分析的难度，提高了检测灵敏度，同时费用相对较低，检测速度更快，具有较好的临床实用价值。利用CGP平台检测患者的*ALK*基因融合状态指导靶向治疗，检测初治患者的*ALK*、*EGFR*或*KRAS*等基因的突变情况提前发现潜在的原发耐药，检测耐药患者的继发*ALK*突变或旁路基因改变选择敏感的下一代ALK抑制剂或旁路通路抑制剂，以及检测患者疗前或治疗过程中*TP53*、*Bim*等基因状态预测和监测患者疗效，可以改善ALK阳性NSCLC患者的预后，降低无效治疗的风险。

虽然*ALK*阳性NSCLC患者的耐药机制逐渐被明确，许多新一代的ALK-TKI和旁路信号通路抑制剂也被针对性地开发并应用于临床，有效地改善了患者的预后。然而，随着新型ALK-TKI的使用，患者的耐药机制正逐渐发生改变。如*ALK* G1269A、L1196M和C1156Y突变常见于Crizotinib耐药患者，而二代ALK-TKI耐药患者主要为*ALK* G1202R突变。与单独Crizotinib治疗患者相比，多重ALK-TKI治疗患者更易出现ALK和旁路信号通路的共激活。ALK抑制剂的序贯使用也会导致肿瘤细胞逐渐累积新的*ALK*突变。上述耐药机制的改变常常会导致现有靶点抑制剂的失效，患者再次出现疾病进展，因而不断需要研发新一代的ALK抑制剂以克服新的耐药机制。所以，研发出更长效的ALK-TKI、使用多靶点抑制剂、联合旁路通路抑制剂或者合理安排用药方案延缓耐药的产生显得十分重要。此外，目前仍有约30%的ALK-TKI耐药患者的耐药机制并不明确，后续还需要对这部分患者进行深入研究，以找到鉴别诊断的生物标志物和研发相应的靶向药。最后，与免疫治疗的PD-L1、TMB以及MSI等检测不同，尚缺少能够广泛应用于临床的靶向治疗疗效标志物。目前仅有少量研究对*ALK*阳性NSCLC患者靶向治疗预后标志物进行了探讨。多数研究纳入的样本量比较有限，现有标志物的预测效能尚不理想，各研究间的结果也存在矛盾，距离临床应用还有较长的距离，后续仍需深入挖掘疾病发生发展以及耐药的机制，扩大样本开发出更多具有预后预测价值的生物标志物，指导靶向治疗的用药方案。
